# Bladder mucosal apoptosis index and quality of life after adjuvant intravesical BCG therapy for non-muscle-invasive bladder cancer: a retrospective propensity-score-matched study

**DOI:** 10.3389/fonc.2026.1856699

**Published:** 2026-07-31

**Authors:** Hui Zhu, ShuBin Tan, XianGen Liu

**Affiliations:** Department of Urology, Affiliated Nantong Hospital 3 of Nantong University, Nantong Third People’s Hospital, Nantong, Jiangsu, China

**Keywords:** apoptosis, Bacillus Calmette-Guérin, non-muscle-invasive bladder cancer, prognostic survival, transurethral resection of bladder tumor

## Abstract

**Objective:**

To evaluate the association of adjuvant intravesical Bacillus Calmette-Guérin (BCG) immunotherapy after transurethral resection of bladder tumor (TURBT) with bladder mucosal apoptosis index, recurrence-related outcomes, progression-free survival, and 12-month quality of life in patients with non-muscle-invasive bladder cancer (NMIBC).

**Methods:**

A retrospective analysis was conducted on 114 NMIBC patients who underwent TURBT at our hospital from January 2022 to December 2024, including 57 patients receiving postoperative intravesical BCG immunotherapy (BCG group) and 57 patients receiving TURBT alone without postoperative adjuvant intravesical therapy (control group). Tumor recurrence rates, progression-free survival (PFS), bladder mucosal apoptosis index, and quality of life (QOL-BREF) scores were compared between groups.

**Results:**

Baseline characteristics of both groups were balanced via propensity score matching (PSM) (P>0.05). The BCG group exhibited a significantly higher bladder mucosal apoptosis index (32. 13 ± 5.61) compared to the control group (18.72 ± 4.29) (P<0.001). Six-month and one-year recurrence rates were markedly lower in the BCG group (2 cases, 3.51%; 8 cases, 14.04%) versus the control group (9 cases, 15.79%; 21 cases, 36.84%) (P<0.001), with median PFS extended to 25 months (control group: 13 months, P<0.001). Pearson correlation analysis showed a positive correlation between apoptosis index and PFS (r=0.61, P<0.001), and multivariable Cox regression further indicated that a higher apoptosis index was independently associated with a lower risk of recurrence or progression. Preoperative QOL-BREF scores showed no significant difference between groups (54. 11 ± 5.96 vs. 54.03 ± 6.27, P>0.05), while postoperative scores were significantly higher in the BCG group (71.95 ± 8.23 vs. 63.47 ± 8.22) (P<0.001).

**Conclusion:**

In this retrospective propensity-score-matched cohort, adjuvant intravesical BCG immunotherapy after TURBT was associated with a higher bladder mucosal apoptosis index, lower recurrence risk, longer progression-free survival, and better 12-month QOL-BREF scores. These findings support the clinical relevance of apoptosis index and QOL assessment in postoperative NMIBC management.

## Introduction

Non-muscle-invasive bladder cancer (NMIBC) is one of the most common malignant tumors in the urinary system, accounting for approximately 70%-80% of newly diagnosed bladder cancer cases, characterized by high recurrence rates and potential progression risk (1, 2). Transurethral resection of bladder tumor (TURBT), as the standard initial treatment for NMIBC, effectively removes visible tumor tissues but is associated with a 30%-80% 5-year recurrence rate, with some patients progressing to muscle-invasive bladder cancer (MIBC), severely impacting prognosis and quality of life. Therefore, optimizing adjuvant therapeutic strategies to reduce recurrence risk and delay disease progression has become a central focus of clinical research.

Since its first reported use as an adjuvant treatment for NMIBC in 1976, intravesical Bacillus Calmette-Guérin (BCG) immunotherapy has been recommended as a standard treatment for intermediate- and high-risk NMIBC in international guidelines. Multiple randomized controlled trials (RCTs) and meta-analyses confirm that BCG instillation significantly reduces tumor recurrence rates (relative risk reduction of 30%-40%) and delays progression to MIBC. However, the precise mechanisms of BCG therapy remain incompletely elucidated, currently believed to involve multiple pathways including induction of local immune responses (e.g., activation of CD4+ T cells, macrophage infiltration), promotion of tumor cell apoptosis, and inhibition of angiogenesis. Notably, responses to BCG treatment vary significantly among patients, with approximately 30%-50% experiencing treatment failure or recurrence, suggesting undiscovered molecular mechanisms or biomarkers (3–5).

Apoptosis, as a critical form of programmed cell death, plays a pivotal role in tumorigenesis, progression, and therapeutic response. Previous studies have demonstrated that BCG may induce tumor cell apoptosis through pathways such as upregulating the Fas/FasL signaling axis and activating caspase cascades. However, large-scale clinical evidence remains lacking regarding the quantitative impact of intravesical BCG immunotherapy on bladder mucosal apoptosis indices and its direct association with patient prognosis. Furthermore, existing research predominantly focuses on endpoint indicators such as tumor recurrence rates and progression-free survival (PFS), with insufficient attention to treatment-related changes in quality of life (6–9).

This study conducted a retrospective propensity-score-matched analysis of 114 patients with NMIBC treated with TURBT at our institution. Given that the clinical efficacy of BCG therapy in intermediate- and high-risk NMIBC has been well established, the present study focused on whether postoperative adjuvant intravesical BCG immunotherapy was associated with measurable changes in bladder mucosal apoptosis index and 12-month QOL-BREF scores, and whether the apoptosis index was correlated with recurrence-related prognosis. These findings may provide complementary real-world evidence for postoperative assessment, response evaluation, and individualized follow-up in NMIBC.

## Methods

### Study population

We retrospectively screened consecutive patients who underwent transurethral resection of bladder tumor (TURBT) at our institution between January 2022 and December 2024. A total of 286 patients were initially identified from the institutional TURBT database. After reviewing pathological reports, operative records, and follow-up data, 54 patients with muscle-invasive bladder cancer or non-urothelial malignancy, 38 patients with prior TURBT or recurrent disease before the index TURBT, 12 patients with concomitant other malignancies, 15 patients with severe cardiac, hepatic, or renal dysfunction, and 27 patients with incomplete follow-up data or missing key clinicopathological information were excluded. Finally, 140 eligible patients with pathologically confirmed non-muscle-invasive bladder cancer (NMIBC) remained for further analysis.

Treatment allocation was not assigned by the investigators but was determined during routine clinical practice according to postoperative clinicopathological risk assessment, physician recommendation, contraindications, patient preference, and shared decision-making. Postoperative intravesical BCG immunotherapy was generally recommended for patients with intermediate- or high-risk NMIBC, especially those with T1 disease, high-grade tumors, carcinoma *in situ* where applicable, multifocal tumors, larger tumor size, or other recurrence/progression risk factors, provided that no contraindications to BCG were present. Patients who received postoperative intravesical BCG immunotherapy were classified as the BCG group. Patients who underwent TURBT alone and did not receive postoperative adjuvant intravesical BCG immunotherapy, pirarubicin, mitomycin C, gemcitabine, or any other adjuvant intravesical therapy during the study-defined treatment period were classified as the control group.

Before propensity-score matching, 68 patients were included in the BCG-treated cohort and 72 patients were included in the non-adjuvant-therapy cohort. After 1:1 propensity-score matching, 114 patients were finally included in the analysis, with 57 patients in the BCG group and 57 patients in the control group.

Inclusion criteria were as follows: ① pathologically confirmed primary papillary Ta or T1 NMIBC, with or without concomitant carcinoma *in situ* (CIS); ② first-time TURBT performed at our institution during the study period; and ③ complete postoperative follow-up data. Exclusion criteria were as follows: ① muscle-invasive bladder cancer, non-urothelial malignancy, or isolated CIS/Tis without concurrent papillary Ta/T1 tumor; ② history of prior TURBT or recurrent bladder cancer before the index surgery; ③ concomitant other malignancies; ④ severe cardiac, hepatic, or renal dysfunction; and ⑤ inability to complete follow-up or missing key clinical data.

### Sample size and propensity-score matching

Because this was a retrospective study, the sample size was determined by the number of eligible patients available in the institutional database during the predefined study period rather than by prospective enrollment. A sample size estimation was performed to evaluate whether the final matched cohort was sufficient to detect a clinically meaningful difference in 1-year recurrence rate between groups. Based on previous studies and historical data from our center, the expected difference in 1-year recurrence rate between the BCG group and the control group was approximately 20%-25%. With α=0.05 and 1-β=0.90, the estimated minimum sample size was approximately 52 patients per group. After 1:1 propensity-score matching, 57 patients were retained in each group, which met the estimated minimum sample size requirement.

Propensity-score matching was performed to reduce baseline imbalance between the BCG and control groups. The variables included in the propensity-score model were age, sex, smoking history, tumor stage, tumor grade, tumor size, multifocality, number of tumors, presence or absence of concomitant carcinoma *in situ* (concomitant CIS), risk classification, performance of second TURBT, and relevant medical history. Use of enhanced cystoscopy, including PDD or NBI, and immediate postoperative intravesical chemotherapy were also recorded and compared between groups. Patients were matched at a 1:1 ratio using the nearest-neighbor method without replacement, with a caliper width of 0.20 of the standard deviation of the logit of the propensity score. Balance between groups after matching was assessed using P values and standardized mean differences.

### Ethical considerations

This retrospective study was conducted in accordance with the Declaration of Helsinki and was reviewed and approved by the Medical Ethics Committee of Nantong Third People’s Hospital Affiliated to Nantong University (Approval No.: 24MLKYW-LC03). All patient data were obtained from the electronic medical record system and were anonymized before analysis. Because this study used de-identified retrospective clinical data and did not involve any additional intervention, follow-up procedure, or risk to patients, the requirement for additional written informed consent was waived by the ethics committee. Patient privacy was strictly protected throughout the study. No potentially identifiable patient images or personal information are presented in this manuscript.

### Grouping and interventions

After propensity-score matching, patients were divided into the BCG group and the control group according to the actual postoperative treatment recorded in the medical records. Patients in the BCG group received postoperative intravesical BCG immunotherapy. The BCG product used in this study was VesiCulture^®^ BCG, a powder for intravesical suspension containing Mycobacterium bovis BCG, Danish strain 1331, live attenuated, 30 mg/vial, manufactured by AJ Vaccines A/S, Denmark. Four vials were used for each instillation, corresponding to a total dose of 120 mg. Before instillation, 120 mg of BCG was dissolved in 50 mL of 0.9% sodium chloride solution. The first instillation was initiated two weeks after TURBT when there was no gross hematuria, urinary tract infection, or other contraindication. The induction phase consisted of weekly instillations for six consecutive weeks, followed by maintenance instillations every two weeks for three cycles and then monthly instillations for one year, with 19 planned instillations in total. Patients in the control group received TURBT alone followed by routine postoperative surveillance. No patient in the control group received postoperative adjuvant intravesical BCG immunotherapy, pirarubicin, mitomycin C, gemcitabine, or other adjuvant intravesical therapy during the study-defined treatment period. Therefore, the control group was defined as the TURBT-alone group rather than an intravesical chemotherapy group. Treatment implementation was reviewed from medical records. The number of actual instillations, induction completion, completion of the institution-defined one-year planned BCG course, adequate BCG therapy, treatment delay, treatment discontinuation, and reasons for discontinuation were recorded. To avoid confusion with the three-year BCG maintenance schedule recommended in guidelines, completion of the “full planned BCG course” in this study was defined as completion of the institution-defined one-year postoperative BCG regimen planned at our center, rather than completion of a three-year maintenance schedule. Induction completion was defined as completion of at least five of the six weekly induction instillations. Completion of the one-year planned BCG course was defined as completion of all planned induction and maintenance instillations within the study-defined one-year treatment period, allowing only short treatment delays for transient symptoms. Adequate BCG therapy was defined as completion of at least five of six induction instillations and at least two maintenance instillations (10–12).

### TURBT procedure and use of enhanced cystoscopy

All patients underwent TURBT by experienced urologists according to standard institutional procedures. The bladder was systematically inspected before resection, including the tumor location, number, size, morphology, and surrounding mucosa. TURBT was mainly performed under white-light cystoscopy. Photodynamic diagnosis (PDD) was not used in either group during the study period. Narrow-band imaging (NBI) was used in selected cases according to equipment availability and the surgeon’s judgment. The use of NBI was recorded and compared between the two groups.

During TURBT, all visible tumors were resected as completely as possible, and deep resection was performed to obtain detrusor muscle when clinically feasible. The tumor base and suspicious surrounding mucosa were additionally treated or sampled when necessary. All resected specimens were submitted for pathological examination to determine tumor stage, grade, and the presence or absence of CIS. Because CIS may occur either as isolated CIS/Tis or as concomitant CIS associated with papillary tumors, the original pathological reports were rechecked for CIS classification. Isolated CIS/Tis was defined as CIS without a concurrent papillary Ta/T1 lesion, whereas concomitant CIS was defined as CIS detected together with papillary Ta or T1 urothelial carcinoma. In the present matched cohort, no isolated CIS/Tis cases were identified. All CIS cases represented concomitant CIS associated with papillary T1 tumors. Therefore, unless otherwise specified, the CIS variable in this study refers to concomitant CIS.

Second TURBT was performed when clinically indicated, including T1 disease, high-grade tumors, absence of detrusor muscle in the initial specimen, suspected incomplete resection, large or multifocal tumors, or suspected residual tumor on early postoperative evaluation. The performance of second TURBT was recorded and included in the baseline comparison between groups.

### Postoperative follow-up protocol

All patients were followed according to the institutional postoperative surveillance protocol for NMIBC. Cystoscopy was scheduled every 3 months during the first year after TURBT and then every 3–6 months according to tumor risk and clinical condition. In this study, recurrence status at 6 months and 12 months was used for the primary recurrence-rate analysis. Urine cytology was performed during follow-up, especially in patients with high-risk disease, CIS, suspicious cystoscopic findings, or unexplained urinary symptoms. Imaging examinations, including urinary ultrasound, computed tomography, or CT urography, were performed when upper urinary tract involvement, disease progression, or metastasis was clinically suspected.

Tumor recurrence was defined as the appearance of a new intravesical bladder tumor lesion after complete TURBT, mainly detected by cystoscopy. Pathological confirmation was obtained when biopsy or repeat resection was performed. Upper urinary tract lesions without concomitant bladder lesions were not counted as bladder recurrence. Disease progression was defined as progression to muscle-invasive bladder cancer, an increase in tumor stage or grade, confirmed upper urinary tract urothelial carcinoma, or the occurrence of regional or distant metastasis. Therefore, if an isolated upper urinary tract lesion had been identified during follow-up, it would have been classified as disease progression rather than bladder recurrence. PFS was defined as the time from the date of TURBT to the first documented bladder recurrence, disease progression, or the last follow-up. Patients without recurrence or progression were censored at the last available follow-up. Follow-up adherence was assessed by reviewing scheduled follow-up visits, cystoscopy records, urine cytology records, imaging records, and readmission or outpatient records.

### Observation indicators

Tumor recurrence rate: Intravesical bladder recurrence was recorded at 6 months and 12 months after TURBT according to the postoperative follow-up protocol described above.

Progression-free survival (PFS): PFS was calculated from the date of TURBT to the first documented intravesical bladder recurrence, disease progression, or the last follow-up. Patients without recurrence or progression were censored at the last available follow-up.

Bladder mucosal apoptosis index: Bladder mucosal apoptosis was evaluated using the terminal deoxynucleotidyl transferase-mediated dUTP nick-end labeling (TUNEL) method. Tissue specimens used for apoptosis assessment were obtained from available paraffin-embedded bladder specimens collected during routine clinical care, including TURBT specimens and clinically indicated follow-up cystoscopic biopsy or repeat resection specimens within 12 months after surgery. No additional biopsy was performed solely for this retrospective study.

When available, non-necrotic tumor tissue or tumor-adjacent bladder mucosa from the tumor base or suspicious mucosal areas was selected for evaluation. Cold-cup biopsy specimens were preferred for follow-up mucosal sampling, whereas TUR specimens were used when biopsy specimens were unavailable. For TUR specimens, areas with obvious necrosis, crush artifact, severe cautery artifact, or thermal damage were excluded to reduce false-positive TUNEL staining caused by electrocautery injury.

All specimens were fixed in 10% neutral buffered formalin, embedded in paraffin, and sectioned at a thickness of 4 μm. TUNEL staining was performed using a commercially available TUNEL assay kit according to the manufacturer’s instructions. Briefly, sections were deparaffinized, rehydrated, treated with proteinase K, incubated with the TUNEL reaction mixture, developed with chromogenic substrate, and counterstained with hematoxylin. Positive and negative controls were included in each staining batch.

TUNEL-positive cells were defined as cells showing brown nuclear staining. For each case, five non-overlapping high-power fields were selected under 400× magnification, avoiding necrotic or thermally damaged areas. At least 500 epithelial or tumor cells were counted when sufficient tissue was available. The apoptosis index was calculated as follows: apoptosis index (%) = number of TUNEL-positive cells/total number of counted cells × 100%. All slides were independently evaluated by two experienced pathologists who were blinded to patient grouping and clinical outcomes. Any inconsistent results were resolved by joint review.

Quality of life score: Quality of life was assessed using the QOL-BREF scale within one week before surgery and at 12 months after surgery. The scale evaluates physical, psychological, social, and environmental aspects of quality of life. Scores were converted to a 0–100 scale, with higher scores indicating better quality of life. The questionnaire was completed under standardized instructions, and the data were checked by two researchers to reduce recording and scoring errors.

Safety assessment: BCG-related adverse events were reviewed from medical records during the treatment and follow-up period, including bladder irritation symptoms, fever, hematuria, urinary tract infection, systemic infection, treatment delay, and treatment discontinuation. Treatment completion was assessed according to the actual number of BCG instillations recorded in the medical records. The relationship between QOL-BREF scores and adverse events or recurrence status was further explored by comparing postoperative QOL-BREF scores between patients with and without BCG-related adverse events and between patients with and without tumor recurrence.

### Statistical methods

Data analysis was performed using SPSS 26.0 software and R software. Continuous variables are presented as mean ± standard deviation or median with interquartile range, as appropriate, and categorical variables are presented as counts and percentages. Between-group comparisons were performed using the independent-samples t-test or Mann–Whitney U test for continuous variables and the chi-square test or Fisher’s exact test for categorical variables.

Propensity-score matching (PSM) was performed to reduce baseline imbalance between the BCG and control groups. The variables included in the propensity-score model were age, sex, smoking history, tumor stage, tumor grade, tumor size, multifocality, number of tumors, presence or absence of CIS, risk classification, performance of second TURBT, relevant medical history, use of enhanced cystoscopy, and immediate postoperative intravesical chemotherapy. Patients were matched at a 1:1 ratio using the nearest-neighbor method without replacement, with a caliper width of 0.20 of the standard deviation of the logit of the propensity score. Balance between groups before and after matching was assessed using standardized mean differences (SMDs), with an SMD <0.10 considered to indicate good balance.

Survival analysis was performed using the Kaplan–Meier method, and differences between groups were compared using the log-rank test. Patients without recurrence or progression at the last follow-up were treated as censored cases. Hazard ratios (HRs) and 95% confidence intervals (CIs) for PFS were estimated using Cox proportional hazards regression. Numbers at risk were displayed below the Kaplan–Meier curves.

The association between bladder mucosal apoptosis index and PFS was first evaluated using Pearson’s correlation analysis. To further assess whether the apoptosis index was independently associated with PFS, multivariable Cox regression analysis was performed after adjustment for clinically relevant oncological factors, including treatment group, tumor stage, tumor grade, concomitant CIS, tumor size, multifocality, number of tumors, risk classification, and second TURBT. A two-sided P value <0.05 was considered statistically significant.

## Results

### General information

During the study period, 286 patients who underwent TURBT were initially identified from the institutional database. After applying the inclusion and exclusion criteria, 140 eligible NMIBC patients remained for propensity-score matching, including 68 patients who received postoperative intravesical BCG immunotherapy and 72 patients who received TURBT alone without postoperative adjuvant intravesical therapy. After 1:1 propensity-score matching, 114 patients were finally included in the analysis, with 57 patients in the BCG group and 57 patients in the control group. Baseline demographic and clinicopathological characteristics are shown in **Table 1**. No statistically significant differences were observed between the two groups in age, sex, smoking history, tumor stage, tumor grade, CIS, tumor size, multifocality, number of tumors, risk classification, second TURBT, use of enhanced cystoscopy, or immediate postoperative intravesical chemotherapy (all P>0.05), indicating that the two groups were generally comparable after matching. See **Table 1**. After rechecking the original pathological reports, no isolated CIS/Tis cases were identified in the matched cohort. Concomitant CIS was observed in 9 of 114 patients (7.89%), including 5 of 57 patients (8.77%) in the BCG group and 4 of 57 patients (7.02%) in the control group. All concomitant CIS cases occurred in patients with T1 disease. Among the 44 patients with T1 disease, concomitant CIS was present in 9 patients (20.45%). In a further descriptive subgroup review, high-grade T1 disease was identified in 29 patients, of whom 9 had concomitant CIS (31.03%), including 5 of 15 patients (33.33%) in the BCG group and 4 of 14 patients (28.57%) in the control group.

**Table 1:** Comparison of baseline demographic and clinicopathological characteristics between the two groups after propensity-score matching.

Characteristic	BCG group	Control group	t/x2	P
Age (years)	60.87 ± 10.78	60.27 ± 10.44	0.302	0.763
Male	30 (52.63)	32 (56.14)	0.141	0.707
Smoking history	24 (42.11)	22 (38.60)	0.146	0.702
Tumor stage			0.148	0.700
Ta	34 (59.65)	36 (63.16)		
T1	23 (40.35)	21 (36.84)		
Tumor grade			0.141	0.707
Low grade	31 (54.39)	33 (57.89)		
High grade	26 (45.61)	24 (42.11)		
Isolated CIS/Tis			0.120	0.729
Concomitant CIS with papillary T1 tumors	5 (8.77)	4 (7.02)		
Concomitant CIS among high-grade T1 cases	5/15 (33.33)	4/14 (28.57)		
Tumor size			0.154	0.695
<3 cm	38 (66.67)	36 (63.16)		
≥3 cm	19 (33.33)	21 (36.84)		
Multifocality			0.036	0.849
Single tumor	35 (61.40)	34 (59.65)		
Multiple tumors	22 (38.60)	23 (40.35)		
Number of tumors			0.058	0.971
1	35 (61.40)	34 (59.65)		
2–3	16 (28.07)	17 (29.82)		
≥4	6 (10.53)	6 (10.53)		
Risk classification			0.038	0.846
Intermediate risk	35 (61.40)	36 (63.16)		
High risk	22 (38.60)	21 (36.84)		
Second TURBT	12 (21.05)	11 (19.30)	0.054	0.817
Use of PDD	0 (0.00)	0 (0.00)	–	–
Use of NBI	7 (12.28)	6 (10.53)	0.086	0.769
Immediate postoperative intravesical chemotherapy	0 (0.00)	0 (0.00)	–	–

CIS, carcinoma *in situ*; TURBT, transurethral resection of bladder tumor; PDD, photodynamic diagnosis; NBI, narrow-band imaging. PDD was not used in either group during the study period. NBI was used only in selected cases and showed no significant difference between groups. Immediate postoperative intravesical chemotherapy was not administered in either group during the study-defined treatment period.

After propensity-score matching, all included baseline variables were well balanced between the two groups. The SMDs for age, sex, smoking history, tumor stage, tumor grade, CIS, tumor size, multifocality, number of tumors, risk classification, second TURBT, NBI use, and immediate postoperative intravesical chemotherapy were all below 0.10, indicating acceptable post-matching balance. See **Supplementary Table 1**.

### Treatment completion and safety

No patient in either group received pirarubicin intravesical instillation during the study-defined treatment period. After rechecking the original instillation records, the planned number of BCG instillations in the BCG group was confirmed to be 19 in the institution-defined one-year regimen. The median actual number of instillations was 17 (interquartile range, 15–19). A total of 55 patients (96.49%) completed the induction phase, 42 patients (73.68%) completed the institution-defined one-year planned BCG course, and 49 patients (85.96%) met the definition of adequate BCG therapy. Compared with the previous version, the completion rate of the full planned BCG course was revised after distinguishing strict completion of all planned instillations from adequate BCG exposure.

BCG-related adverse events were generally mild and manageable. Bladder irritation symptoms were observed in 12 patients (21.05%), transient fever in 6 patients (10.53%), hematuria in 5 patients (8.77%), and urinary tract infection in 3 patients (5.26%). No patient developed severe systemic BCG infection. Treatment delay occurred in 9 patients (15.79%), mainly because of transient bladder irritation symptoms, fever, hematuria, scheduling adjustment, or temporary urinary symptoms. Fifteen patients (26.32%) did not complete all 19 planned instillations. The reasons for non-completion included adverse events in 5 patients (8.77%), tumor recurrence before completion in 4 patients (7.02%), and patient preference or logistical reasons in 6 patients (10.53%). No treatment delay or discontinuation related to intravesical therapy occurred in the control group because patients in this group did not receive postoperative adjuvant intravesical therapy. See **Table 2**.

**Table 2:** Treatment completion and BCG-related adverse events.

Variable	BCG group (n=57)	Control group (n=57)
Postoperative BCG immunotherapy	57 (100.00)	0 (0.00)
Pirarubicin intravesical instillation	0 (0.00)	0 (0.00)
Planned number of BCG instillations	19	Not applicable
Actual number of BCG instillations, median (IQR)	17 (15–19)	Not applicable
Completion of induction BCG	55 (96.49)	Not applicable
Completion of institution-defined one-year planned BCG course	42 (73.68)	Not applicable
Adequate BCG therapy	49 (85.96)	Not applicable
Non-completion of one-year planned BCG course	15 (26.32)	Not applicable
Non-completion due to adverse events	5 (8.77)	Not applicable
Non-completion due to recurrence before course completion	4 (7.02)	Not applicable
Non-completion due to patient preference or logistical reasons	6 (10.53)	Not applicable
Bladder irritation symptoms	12 (21.05)	Not applicable
Fever	6 (10.53)	Not applicable
Hematuria	5 (8.77)	Not applicable
Urinary tract infection	3 (5.26)	Not applicable
Severe systemic BCG infection	0 (0.00)	Not applicable
Treatment delay	9 (15.79)	Not applicable
Treatment discontinuation due to adverse events	5 (8.77)	Not applicable

BCG, Bacillus Calmette-Guérin; IQR, interquartile range. Completion of the institution-defined one-year planned BCG course was defined as completion of all planned induction and maintenance instillations within the study-defined one-year treatment period. This definition differs from the three-year BCG maintenance schedule recommended in international guidelines. Adequate BCG therapy was defined as completion of at least five of six induction instillations and at least two maintenance instillations. Reasons for non-completion were mutually exclusive. BCG-related adverse events were reviewed from medical records, and individual patients could have more than one adverse event. The control group did not receive postoperative adjuvant intravesical therapy; therefore, BCG-related adverse events and BCG treatment completion were not applicable.

### Follow-up completion and recurrence assessment

The median follow-up duration was 25 months (IQR, 18–30 months) in the BCG group and 24 months (IQR, 18–29 months) in the control group. All included patients had available 12-month recurrence assessment data. Scheduled cystoscopic follow-up within the predefined follow-up window was completed by 55 patients (96.49%) in the BCG group and 54 patients (94.74%) in the control group. Delayed follow-up occurred in 2 patients (3.51%) in the BCG group and 3 patients (5.26%) in the control group, but their recurrence status could still be confirmed through subsequent cystoscopy, outpatient records, or readmission records. Urine cytology was performed during follow-up when clinically indicated, particularly in patients with high-risk features, CIS, suspicious cystoscopic findings, or urinary symptoms. After rechecking cystoscopy, pathology, urine cytology, and imaging records, no patient was found to have an isolated upper urinary tract lesion without concomitant bladder recurrence during the follow-up period. Among patients with recurrence, pathological confirmation was obtained in all recurrent cases when biopsy or repeat resection was performed. The follow-up procedures and adherence were comparable between the two groups.

### Cell apoptosis

**Figure 1:**
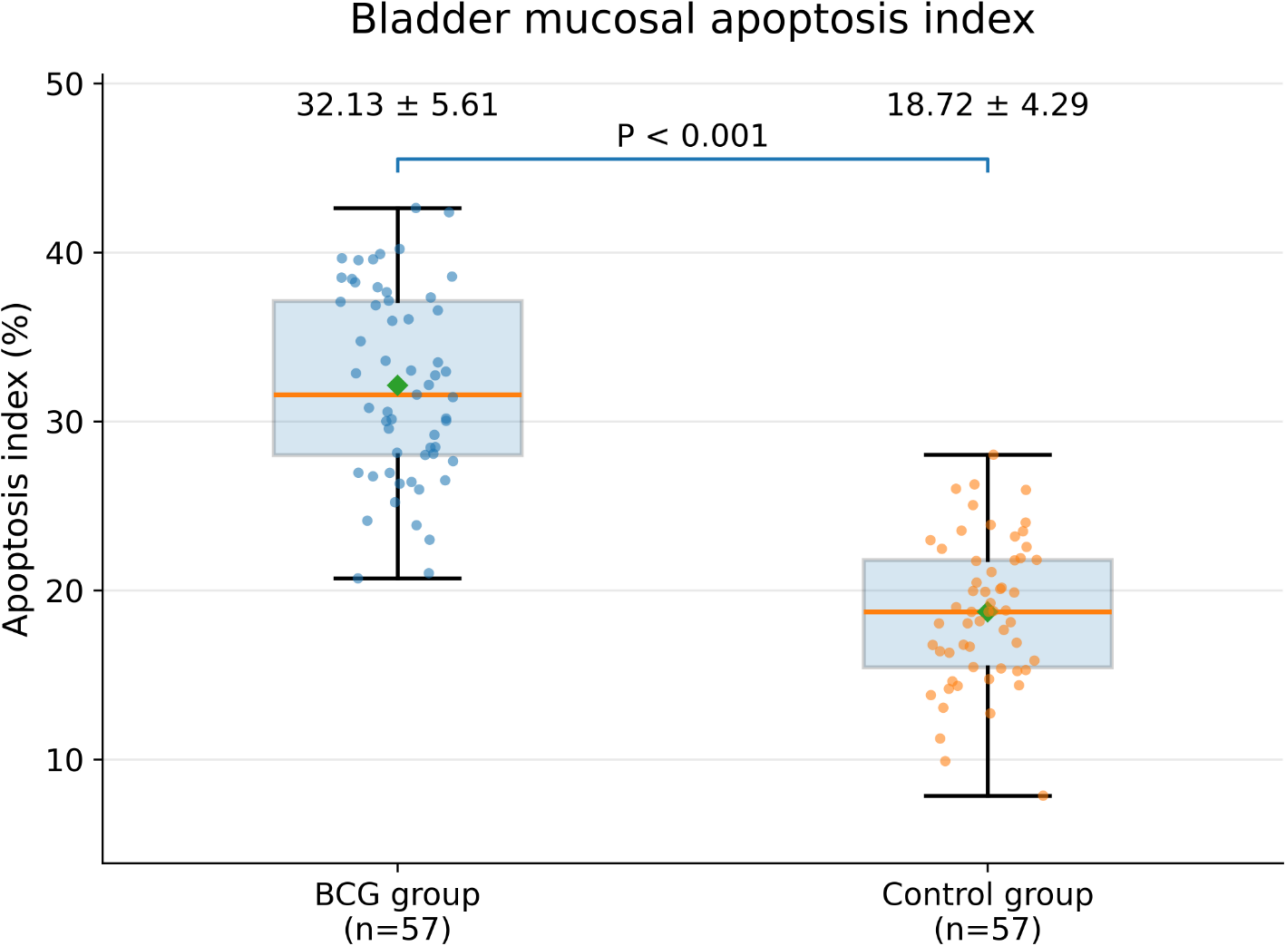
Comparison of bladder mucosal apoptosis index between the BCG group and the TURBT-alone control group. Data are shown as box-and-whisker plots overlaid with jittered individual data points. The center line indicates the median, boxes indicate the interquartile range, whiskers indicate the data range, and diamonds indicate the mean. The bladder mucosal apoptosis index was significantly higher in the BCG group than in the control group. BCG, Bacillus Calmette-Guérin; TURBT, transurethral resection of bladder tumor.

The bladder mucosal apoptosis index in the BCG group was 32.13 ± 5.61, which was significantly higher than that in the control group (18.72 ± 4.29, P < 0.001). See **Table 3** and **Figure 1**.

**Table 3:** Comparison of bladder mucosal apoptosis index between the two groups.

Variable	BCG group	Control group	*t*	*P*
Number of Cases	57	57	–	–
Apoptosis index of bladder mucosa cells	32. 13 ± 5.61	18.72 ± 4.29	14.336	<0.001

### Prognosis and survival

**Figure 2:**
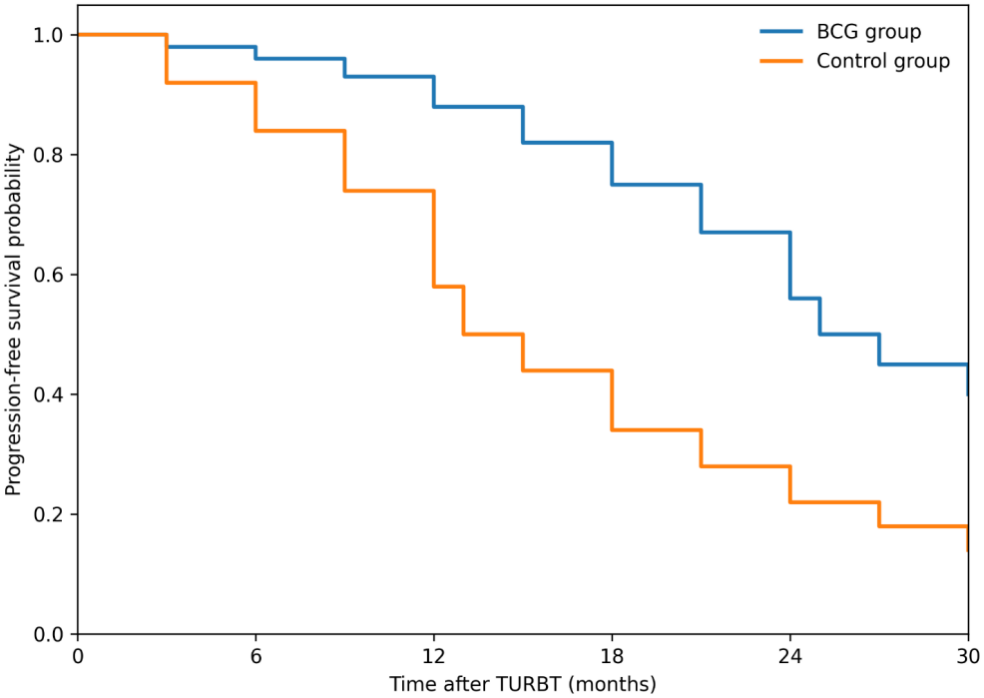
Kaplan–Meier curves for progression-free survival in the BCG and control groups. The Kaplan–Meier curves show progression-free survival after TURBT in the BCG group and control group. Patients without recurrence or progression at the last follow-up were censored. Numbers at risk are shown below the curves. The median PFS was 25 months in the BCG group and 13 months in the control group. Cox proportional hazards regression showed that BCG immunotherapy was associated with a lower risk of recurrence or progression than TURBT alone (HR = 0.46, 95% CI: 0.28–0.76, P = 0.002).

The 6-month and 1-year recurrence rates in the BCG group were significantly lower than those in the control group (2 cases [3.51%] and 8 cases [14.04%] vs. 9 cases [15.79%] and 21 cases [36.84%], respectively; P<0.001). Kaplan–Meier analysis showed that PFS was significantly longer in the BCG group than in the control group. The median PFS was 25 months in the BCG group and 13 months in the control group. The log-rank test showed a significant between-group difference (P<0.001). In Cox proportional hazards regression analysis, postoperative intravesical BCG immunotherapy was associated with a reduced risk of recurrence or progression compared with TURBT alone (HR = 0.46, 95% CI: 0.28–0.76, P = 0.002). Patients without recurrence or progression at the last follow-up were treated as censored cases. The Kaplan–Meier curves and numbers at risk are shown in **Figure 2**. See **Tables 4**, **5** and **Figure 2**.

**Table 4:** Comparison of recurrence rates within one year between the two groups.

Variable	BCG group	Control group	x2	*P*
Number of				
–	57	57	–	–
Cases				
Recurrence Half a year	3.51%	15.79%	4.930	<0.05
rate One year	14.04%	36.84%	7.816	<0.001

**Table 5:** Comparison of progression-free survival between the two groups.

Variable	BCG group	Control group	P
Number of Cases	57	57	–
Median PFS (month)	25	13	<0.001

### Correlation

Pearson’s correlation analysis showed a positive correlation between bladder mucosal apoptosis index and PFS (r=0.61, P<0.001). To further assess whether the apoptosis index was independently associated with PFS, multivariable Cox regression analysis was performed after adjustment for treatment group, tumor stage, tumor grade, CIS, tumor size, multifocality, number of tumors, risk classification, and second TURBT. The results showed that a higher apoptosis index was independently associated with a lower risk of recurrence or progression (adjusted HR per 5% increase=0.74, 95% CI: 0.61–0.89, P = 0.002). This suggests that the apoptosis index may be associated with improved prognosis independent of major clinicopathological factors. See **Table 6**.

**Table 6:** Association between bladder mucosal apoptosis index and PFS.

Variable	HR	95% CI	P
Apoptosis index, per 5% increase	0.74	0.61–0.89	0.002
BCG treatment	0.48	0.29–0.80	0.004
T1 stage	1.42	0.82–2.47	0.210
High-grade tumor	1.56	0.89–2.75	0.118
Concomitant CIS	1.39	0.61–3.17	0.431
Tumor size ≥3 cm	1.34	0.78–2.31	0.286
Multiple tumors	1.47	0.86–2.52	0.160
High-risk classification	1.62	0.92–2.87	0.095
Second TURBT	0.91	0.48–1.72	0.771

### Quality of life

**Figure 3:**
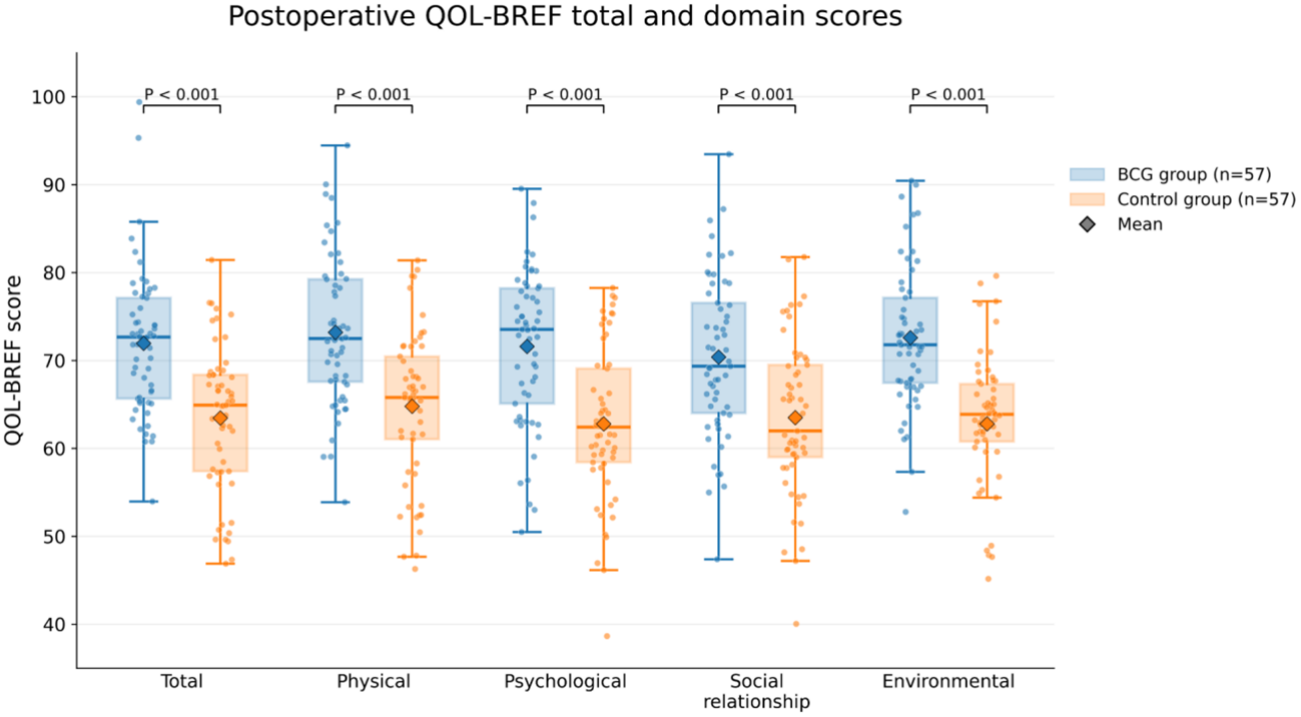
Comparison of postoperative QOL-BREF total and domain-specific scores between the BCG group and the TURBT-alone control group. Data are shown as box-and-whisker plots overlaid with jittered individual data points. The center line indicates the median, boxes indicate the interquartile range, whiskers indicate the data range, and diamonds indicate the mean. The postoperative total, physical, psychological, social relationship, and environmental domain scores were significantly higher in the BCG group than in the control group. BCG, Bacillus Calmette-Guérin; QOL-BREF, World Health Organization Quality of Life-BREF scale; TURBT, transurethral resection of bladder tumor.

No statistically significant difference in total QOL-BREF score was observed between the BCG group and the TURBT-alone control group before surgery (54.11 ± 5.96 vs. 54.03 ± 6.27, P = 0.945). **Table 7** compares QOL-BREF total and domain scores between the BCG group and the control group, rather than between patients with and without BCG-related adverse events. At 12 months after surgery, the total QOL-BREF score was higher in the BCG group than in the control group (71.95 ± 8.23 vs. 63.47 ± 8.22, P<0.001). The postoperative physical domain score was also higher in the BCG group (73.20 ± 8.50 vs. 64.80 ± 8.60, P<0.001), but this result should be interpreted as a between-group difference in postoperative QOL, not as evidence that BCG directly improved physical symptoms. Exploratory analysis related to BCG adverse events and recurrence status is presented separately in **Table 8**. See **Tables 7**, **8**, and **Figure 3**.

**Table 7:** Comparison of QOL-BREF total and domain scores between the BCG group and the TURBT-alone control group.

Variable	BCG group (n=57)	Control group (n=57)	*t*	*P*
Total score before surgery	54.11 ± 5.96	54.03 ± 6.27	0.070	0.945
Physical domain before surgery	52.80 ± 6.40	52.50 ± 6.70	0.244	0.808
Psychological domain before surgery	53.90 ± 6.80	53.60 ± 6.50	0.241	0.810
Social relationship domain before surgery	55.10 ± 7.20	54.90 ± 7.00	0.150	0.881
Environmental domain before surgery	54.60 ± 6.90	55.00 ± 6.60	0.316	0.753
Total score after surgery	71.95 ± 8.23	63.47 ± 8.22	5.504	<0.001
Physical domain after surgery	73.20 ± 8.50	64.80 ± 8.60	5.244	<0.001
Psychological domain after surgery	71.60 ± 8.90	62.80 ± 8.70	5.340	<0.001
Social relationship domain after surgery	70.40 ± 9.10	63.50 ± 8.80	4.116	<0.001
Environmental domain after surgery	72.60 ± 8.00	62.80 ± 8.50	6.331	<0.001

QOL-BREF, World Health Organization Quality of Life-BREF scale; BCG, Bacillus Calmette-Guérin; TURBT, transurethral resection of bladder tumor. This table compares the BCG group with the TURBT-alone control group and does not compare patients with and without BCG-related adverse events.

**Table 8:** Exploratory analysis of postoperative QOL-BREF score according to BCG-related adverse events and recurrence status.

Variable	Number of patients	Postoperative QOL-BREF score	*t*	*P*
BCG group with adverse events	18	68.37 ± 8.21	2.440	0.018
BCG group without adverse events	39	73.84 ± 7.69		
Patients with recurrence within 12 months	29	64.18 ± 8.43	4.839	<0.001
Patients without recurrence within 12 months	85	72.56 ± 7.82		

QOL-BREF, World Health Organization Quality of Life-BREF scale. BCG-related adverse events included bladder irritation symptoms, fever, hematuria, urinary tract infection, treatment delay, and treatment discontinuation.

To further explore factors associated with postoperative quality of life, patients were stratified according to adverse events and recurrence status. In the BCG group, patients without BCG-related adverse events had higher postoperative QOL-BREF scores than those with adverse events (73.84 ± 7.69 vs. 68.37 ± 8.21, P = 0.018). In the overall matched cohort, patients without tumor recurrence within 12 months had higher postoperative QOL-BREF scores than those with recurrence (72.56 ± 7.82 vs. 64.18 ± 8.43, P<0.001). See **Table 8**.

## Discussion

This study was not intended to re-establish the already recognized efficacy of BCG therapy in NMIBC, but rather to evaluate its association with bladder mucosal apoptosis index, recurrence-related prognosis, and patient-reported QOL in a real-world propensity-score-matched cohort. The results showed that patients receiving adjuvant intravesical BCG immunotherapy after TURBT had a higher bladder mucosal apoptosis index, lower recurrence rates, longer PFS, and better 12-month QOL-BREF scores than patients treated with TURBT alone. These findings suggest that apoptosis index and QOL assessment may provide additional information beyond conventional recurrence and survival outcomes when evaluating postoperative management in NMIBC.

First, after balancing the baseline data of the two groups using propensity score matching (PSM), no statistical differences were observed, ensuring the reliability of the study results. This approach excluded the interference of confounding factors such as age, gender, tumor stage, and tumor size on the efficacy assessment, aligning with the design specifications of retrospective studies. The bladder mucosal apoptosis index in the BCG group was significantly higher than that in the control group, suggesting that BCG may exert an antitumor effect by inducing tumor cell apoptosis. Previous studies have confirmed that BCG can trigger the tumor cell apoptosis program through mechanisms such as activating the Fas/FasL death receptor pathway, promoting caspase-3/7 enzymatic cleavage cascades, and releasing cytochrome C via the mitochondrial pathway (13–15). In this study, a significant positive correlation was observed between the apoptosis index and PFS (r = 0.61), further supporting the notion that apoptosis upregulation is a key pathway in the antitumor effect of BCG. This may reduce the risk of recurrence and prolong survival by clearing residual tumor cells, inhibiting the proliferation of tumor stem cells, and reducing the formation of micrometastases.

The 6-month and 1-year recurrence rates in the BCG group were significantly lower than those in the control group, and the median PFS was prolonged to 25 months. This therapeutic advantage may stem from the local immune response induced by BCG. As an attenuated live vaccine of mycobacteria, BCG can activate immune cells in the lamina propria of the bladder mucosa, including dendritic cells, macrophages, and CD4+/CD8+ T lymphocytes, promoting Th1-type immune responses and the secretion of cytokines (such as IL- 12 and IFN-γ), thereby forming an antitumor immune microenvironment. This immune activation effect not only directly kills tumor cells but also induces immune memory, inhibiting tumor recurrence and progression (16–19). Additionally, BCG instillation may further block tumor nutrient supply and metastatic pathways by altering the phenotype of tumor-associated fibroblasts, inhibiting the expression of angiogenic factors (such as VEGF), and regulating extracellular matrix remodeling, thereby prolonging PFS (20, 21).

The CIS distribution in the present cohort also requires clarification. After pathological rechecking, no isolated CIS/Tis cases were identified, and all nine CIS cases represented concomitant CIS associated with papillary T1 tumors. The overall proportion of concomitant CIS was 7.89% in the matched cohort, which appears slightly lower than the approximately 10% prevalence of CIS generally reported in NMIBC populations. However, when the denominator was restricted to T1 disease, concomitant CIS was observed in 20.45% of T1 cases. Moreover, among patients with high-grade T1 disease, the proportion of concomitant CIS was 31.03%, which falls within the 20%–40% range reported for high-grade T1 cohorts. Therefore, the apparently low overall CIS proportion in our cohort is partly attributable to the inclusion of both Ta and T1 papillary NMIBC patients and the absence of isolated CIS/Tis as a separate enrollment category. In addition, patients with prior TURBT or recurrent bladder cancer were excluded, which may have reduced the proportion of CIS-enriched high-risk cases. From a diagnostic perspective, TURBT was mainly performed under white-light cystoscopy, PDD was not used, NBI was used only in selected cases, and systematic mapping biopsies were not routinely performed; therefore, small or flat CIS lesions may have been underdetected. These factors should be considered when interpreting CIS-related findings, and future prospective studies using standardized enhanced cystoscopy and biopsy protocols are needed to more accurately evaluate CIS burden in NMIBC, particularly in T1 and high-grade T1 disease (22). The BCG treatment completion rate also requires cautious interpretation. After rechecking the original instillation records, the completion rate of the institution-defined one-year planned BCG course was revised from 85.96% to 73.68%, whereas 85.96% represented adequate BCG therapy rather than strict completion of all planned instillations. This distinction is important because the “full planned BCG course” in the present study referred to the one-year postoperative regimen used at our center, not to the full three-year maintenance schedule recommended in international guidelines. According to the EAU guideline, a complete BCG schedule includes six weekly induction instillations followed by three-weekly maintenance instillations at 3, 6, 12, 18, 24, 30, and 36 months; however, treatment discontinuation is common in clinical practice, and completion of long-term maintenance is often limited by local or systemic adverse events, recurrence, patient preference, and logistical factors (23). In the present cohort, the relatively high one-year completion rate may be partly explained by several factors. First, patients with contraindications to BCG, severe organ dysfunction, incomplete follow-up, or missing key treatment records were excluded, which may have selected patients with relatively better treatment tolerance and adherence. Second, this was a single-center cohort with standardized postoperative education, scheduled follow-up reminders, and prompt management of transient bladder irritation symptoms, fever, hematuria, or urinary tract infection. Third, no severe systemic BCG infection occurred, and most adverse events were mild and manageable. Finally, the present completion rate reflects a one-year institutional regimen rather than long-term guideline-defined three-year maintenance; therefore, it should not be directly compared with three-year maintenance completion rates reported in guideline-based cohorts. This issue has been clarified in the revised manuscript, and the relatively high completion rate should be interpreted with caution.

The QOL findings should be interpreted cautiously. In **Table 7**, the QOL-BREF results represent comparisons between the BCG group and the TURBT-alone control group, rather than comparisons between patients with and without BCG-related adverse events. Although the postoperative physical domain score was higher in the BCG group, this finding does not necessarily indicate that BCG directly improved physical symptoms. A more clinically reasonable interpretation is that patients in the BCG group had better tumor control and fewer recurrence-related symptoms during follow-up, which may have contributed to better overall postoperative QOL at 12 months. At the same time, the exploratory analysis in **Table 8** showed that BCG-treated patients with adverse events had lower postoperative QOL-BREF scores than those without adverse events, suggesting that BCG-related adverse events may negatively affect QOL. Therefore, the QOL results should be understood as the combined effect of tumor control, treatment tolerability, and postoperative recovery, rather than as a direct physical benefit of BCG therapy. These findings are consistent with previous reports indicating that postoperative QOL in NMIBC is influenced by recurrence risk, surveillance burden, intravesical treatment-related symptoms, and patient tolerance (24–27).

The therapeutic landscape of NMIBC is also evolving, particularly for patients with BCG-unresponsive high-risk disease and CIS-containing tumors. For these patients, bladder-preserving strategies have expanded beyond repeat BCG alone and include systemic immune checkpoint inhibition, intravesical gene therapy, IL-15-based immunotherapy combined with BCG, sequential intravesical chemotherapy such as gemcitabine/docetaxel, and novel intravesical drug-delivery systems. These emerging options do not diminish the established role of BCG as first-line adjuvant therapy for eligible intermediate- and high-risk NMIBC, but they highlight the need to identify patients who are most likely to benefit from BCG, to monitor treatment tolerance, and to incorporate patient-centered outcomes such as QOL into postoperative assessment. In this context, the apoptosis index and QOL-BREF findings in the present study may provide complementary information for future response evaluation and individualized treatment selection (28–31).

Additionally, this study has several limitations. First, this was a single-center retrospective study, and treatment allocation was based on routine clinical risk assessment, physician recommendation, patient preference, and shared decision-making rather than random assignment. Although propensity-score matching was used to reduce baseline imbalance between groups, residual selection bias and unmeasured confounding factors may still exist, which limits the strength of causal inference. Second, TURBT was mainly performed under white-light cystoscopy. In addition, although thermally damaged areas were excluded from TUNEL evaluation, the retrospective use of available TURBT or biopsy specimens may still introduce some heterogeneity in specimen source and handling. Although PDD was not used in either group and NBI use was comparable after matching, the possibility of missed small lesions or CIS cannot be completely excluded. Third, recurrence assessment was based on a defined postoperative follow-up protocol, and all included patients had available 12-month recurrence data. However, because of the retrospective design, minor variations in follow-up timing and examination selection may still have introduced surveillance or detection bias. Fourth, although the overall sample size of 114 cases met the estimated requirement, the statistical power may still be insufficient for subgroup analyses, such as analyses stratified by tumor stage or grade. Therefore, subgroup findings should be interpreted cautiously. Fifth, the current follow-up duration mainly reflects short-term recurrence and survival outcomes, and is insufficient for evaluating long-term outcomes such as 5-year disease-free survival, overall survival, and late recurrence. Extended follow-up is required to comprehensively assess the long-term benefits and potential risks of BCG therapy. Finally, this study compared postoperative intravesical BCG immunotherapy with TURBT alone without adjuvant intravesical therapy, but did not include active comparator groups such as pirarubicin, mitomycin C, gemcitabine, or other intravesical chemotherapy regimens. Future multicenter, prospective, multi-arm studies are needed to compare the relative efficacy and safety of different postoperative intravesical treatment strategies.

Future research should focus on conducting prospective, multicenter, randomized controlled trials with stricter inclusion and exclusion criteria, blinded assessment, and standardized operating procedures to reduce bias and enhance the level of evidence. By integrating multi-omics technologies (such as transcriptomics, proteomics, and metabolomics), a deeper understanding of the molecular mechanisms underlying BCG’s antitumor effects can be achieved. This includes exploring the regulatory roles of apoptosis-related pathways (such as Fas/FasL and caspase cascades), immune microenvironment markers (such as PD-L1 expression and T-cell infiltration density), and tumor stem cell characteristics in the response to BCG therapy, with the aim of constructing a predictive model to screen patients who are most likely to benefit and enabling individualized treatment. Meanwhile, extending the follow-up period to more than 5 years is essential to systematically evaluate the long-term survival benefits, risk of late recurrence, and potential late-onset adverse effects (such as tuberculosis infection and bladder contraction) of BCG therapy, providing more comprehensive evidence-based support for clinical decision-making.

In conclusion, adjuvant intravesical BCG immunotherapy after TURBT was associated with a higher bladder mucosal apoptosis index, reduced recurrence risk, prolonged PFS, and better 12-month QOL-BREF scores in patients with NMIBC. In the context of an evolving NMIBC treatment landscape, apoptosis index and QOL assessment may provide complementary information for postoperative evaluation and future individualized treatment selection.

## Data Availability

The original contributions presented in the study are included in the article/**Supplementary Material**. Further inquiries can be directed to the corresponding author.

## Supplementary material

Supplementary Table 1 Standardized Mean Differences Before and After Propensity-Score Matching.
